# Long noncoding RNA XIST promotes malignancies of esophageal squamous cell carcinoma via regulation of miR-101/EZH2

**DOI:** 10.18632/oncotarget.18638

**Published:** 2017-06-27

**Authors:** Xiaoliang Wu, Xiaoxiao Dinglin, Xing Wang, Wen Luo, Qi Shen, Yong Li, Ling Gu, Qianghua Zhou, Haotu Zhu, Yanjie Li, Chaodi Tan, Xianzi Yang, Zhenfeng Zhang

**Affiliations:** ^1^ Department of Oncology, Guizhou Provincial People's Hospital, Guiyang, China; ^2^ State Key Laboratory of Oncology in South China & Collaborative Innovation Center for Cancer Medicine, Sun Yat-sen University Cancer Center, Guangzhou, China; ^3^ Department of Medical Oncology, Sun Yat-sen Memorial Hospital, Sun Yat-sen University, Guangzhou, China; ^4^ Puer University, Puer, China; ^5^ The Third Affiliated Hospital of Sun Yat-sen University, Guangzhou, China; ^6^ Sun Yat-sen University, Guangzhou, China; ^7^ Affiliated Cancer Hospital & Institute of Guangzhou Medical University, Guangzhou, China; ^8^ Department of Radiology, The Second Affiliated Hospital of Guangzhou Medical University, Guangzhou, China

**Keywords:** long non-coding RNA, XIST, EZH2, esophageal squamous cell carcinoma, EMT

## Abstract

The long non-coding RNA XIST is a long non-coding RNA that associates with polycomb repressive complex 2 to regulate X-chromosome inactivation in female mammals. The biological roles as well as the underlying mechanisms of XIST in esophageal squamous cell carcinoma remained yet to be solved. Our data indicated that XIST was significantly upregulated in esophageal squamous cancerous tissues and cancer cell lines, as compared with that in the corresponding non-cancerous tissues and immortalized normal squamous epithelial cells. High XIST expression predicted poor prognosis of esophageal squamous cancer patients. Lentivirus mediated knockdown of XIST inhibited proliferation, migration and invasion of esophageal squamous cancer cells *in vitro* and suppressed tumor growth *in vivo*. Knockdown of XIST resulted in elevated expression of miR-101 and decreased expression of EZH2. Further analysis showed that XIST functioned as the competitive endogenous RNA of miR-101 to regulate EZH2 expression. Moreover, enforced expression of EZH2 significantly attenuated the anti-proliferation activity upon XIST knockdown. Conclusively, XIST plays an important role in malignant progression of ESCC via modulation of miR-101/EZH2 axis.

## INTRODUCTION

Esophageal cancer is the eighth most common cancer diagnosed worldwide [1, 2] and is the top five leading cause of cancer-related deaths in China [2]. The esophageal squamous cell carcinoma (ESCC), the major histological subtype of esophageal cancer [3], especially in the Eastern countries [2, 3]. Due to absence of clinical symptoms during disease initiation, the majority of ESCC patients were diagnosed at late stage and thus the prognosis is extremely poor [4], with the overall median survival less than 12 months for advanced patients [5–7]. Aberrant proliferation and distant metastasis are vital steps in tumor development and key determinants of aggressive phenotypes [8, 9]. Because alternatives for precise prediction of prognosis and tumor metastasis after resection of primary lesions in ESCC patients still lack, it is therefore imperative to identify new molecules associated with this deadliest form of cancer.

Long non-coding RNAs (lncRNAs) have emerged as key regulators of many biological processes [10–13], which exquisitely regulate the information flow, whereby the macromolecules including protein complex, genes and chromosomes are assembled in an appropriate and proper manner [10]. LncRNA-based mechanisms have been involved in cell fates control, and their dysregulation have been shown to be associated with several human diseases including cancer [14–16]. The lncRNA XIST (X-inactive specific transcript) is necessary for X-chromosome inactivation in female mammals [12, 13, 17]. Recruitment of the polycomb repressive complex 2 (PRC2) underlies XIST-mediated repression of the entire chromosome and the maintenance of silent state [18, 19]. More recently, upregulation of XIST was reported to be associated with overexpression of EZH2 [20], a key component of PRC2 [21] and to act as an adverse prognosis indicator of gastric cancer patients. Overexpression of XIST has been observed in non-small cell lung cancer [22] and glioblastoma [23]. On the other hand, deletion of XIST in the mice resulted in a highly aggressive myeloproliferative neoplasm with 100% penetrance, indicating tumor-suppressive effects of XIST [15]. However, the exact roles and the underlying mechanisms of XIST in ESCC remained yet to be solved.

## RESULTS

### XIST is upregulated in ESCC and predicts a poor prognosis of ESCC patients

**Figure 1:**
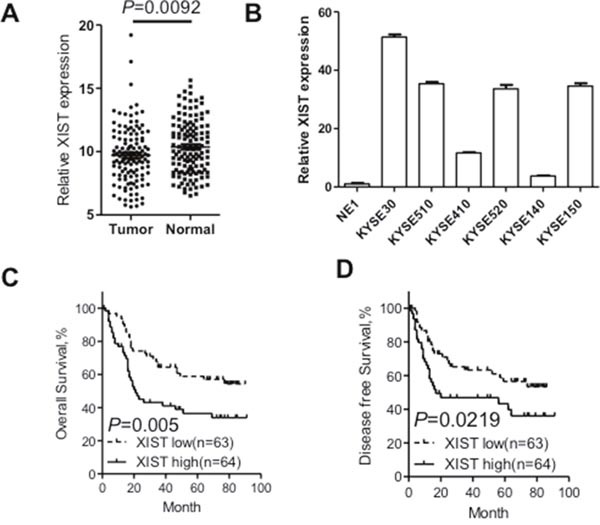
XIST was overexpressed in ESCC tissues and correlates with adverse prognosis of ESCC patients **(A)** Relative XIST expression in ESCC tissues (n=127) compared with corresponding adjacent normal tissues (n=127). XIST expression was examined by qRT-PCR and normalized to GAPDH expression. Results were presented as Δcycle threshold (ΔCt) in tumor tissues relative to normal tissues. **(B)** Expression of XIST in ESCC cell lines (KYSE30, KYSE510, KYSE410, KYSE520, KYSE140 and KYSE150) compared with that of the immortalized esophageal epithelial cell line NE1, data was presented as expression fold change relative to NE1. **(C)** ESCC patients were assigned to high XIST group and low XIST group according to the fold change of XIST in tumor tissues compared with that in normal tissues. Kaplan–Meier curves indicate patients with high-level XIST expression (n=64) showed reduced overall survival time compared with patients with low-level XIST expression (n=63) (*P*=0.005, log-rank test). **(D)** Kaplan–Meier curves indicate patients with high-level XIST expression showed reduced disease-free survival time compared with patients with low-level XIST expression (*P*=0.0219, log-rank test). Error bars: mean ± SD, n = 3. *P < 0.05 versus control.

To explore the biological roles of XIST in ESCC, the expression level of XIST was detected in 127 paired cancer tissues and corresponding adjacent non-tumorous tissues by qRT-PCR. Expression of XIST was significantly upregulated in cancer tissues compared with that in non-tumorous tissues (Figure 1A). We then detected expression of XIST in a panel of ESCC cancer cells including KYSE30, KYSE510, KYSE410, KYSE520, KYSE140 and KYSE150 and one immortalized normal epithelial cells (NE1). Figure 1B showed that expression of XIST in cancer cell lines was remarkably upregulated compared with that in NE1 cells, which was in concordance with that in patient samples. The patient cohort was then divided into high-level group and low-level group with the median expression level serving as the cutoff value. Correlation between XIST expression level and clinicopathological parameters was summarized in Table 1. Additionally, we tested whether expression of XIST could predict prognosis of ESCC through Kaplan-Meier analysis and log-rank tests. Our results showed that patients with high XIST level had a significantly shortened overall survival as well as disease-free survival than those with low XIST level (Figure 1C and 1D). Further univariate and multivariate analyses were performed to evaluate whether the expression of XIST was an independent prognostic factor for ESCC. As shown in Table 2, univariate analysis indicated that tumor size (*P* = 0.019), differentiation state (*P* = 0.048), TNM stage (*P* = 0.000) and XIST expression level (*P* = 0.005) were significantly associated with overall survival of ESCC patients. However, multivariate analysis using the Cox proportional hazards model showed that only TNM stage (*P* = 0.000) and XIST expression level (*P* = 0.001) were independent prognostic factors for ESCC patients. Collectively, XIST is upregulated in ESCC tumor tissues and acts as an independent prognosis predictor for ESCC.

**Table 1:** The correlation between clinicopathological parameters and XIST expression

	XIST expression		*P*
	Low, n (%)	High, n (%)	
Age			
< 60	32 (45.1)	39(54.9)	0.286
≥ 60	31(55.4)	25(44.6)	
Gender			
Male	50(52.1)	46(47.9)	0.410
Female	13(41.9)	18(58.1)	
Alcohol consumption			
Ever and current	23(50.0)	23(50.0)	1.000
Never	40(49.4)	41(50.6)	
Smoking status			
Ever and current	40(54.8)	33(45.2)	0.210
Never	23(42.6)	31(57.4)	
Tumor size			
< 5cm	56(51.9)	52(48.1)	0.320
≥ 5cm	7(36.8)	12(63.2)	
Differentiation status			
Well or Moderate	49(49.0)	51(51.0)	0.831
Poor	14(51.9)	13(48.1)	
TNM stage			0.596
I-II	34(52.3)	31(47.7)	
III	29(46.8)	33(53.2)	

**Table 2:** Univariate and multivariate analyses of various potential prognostic factors in ESCC patients

	Univariate analysis		Multivariate analysis	
	HR(95% CI)	*P*	HR(95% CI)	*P*
Age (< 60/≥ 60)	1.51(0.93-2.47)	0.099	-	-
Gender (Male/Female)	0.81(0.47-1.40)	0.450	-	-
Alcohol (Never/Ever)	0.82(0.57-1.57)	0.824	-	-
Smoke (Never/Ever)	0.97(0.59-1.60)	0.898	-	-
Tumor size(≥ 5cm/< 5 cm)	2.09(1.13-3.85)	0.019*	1.57(0.84-2.93)	0.155
Differentiation(Moderate/Poor, Well)	1.74(1.01-3.01)	0.048*	1.54(0.89-2.67)	0.122
TNM Stage(III/I-II)	3.74(2.18-6.40)	0.000*	3.91(2.25-6.78)	0.000*
XIST (High/Low)	2.06(1.25-3.40)	0.005*	2.40(1.44-4.01)	0.001*

HR: hazard ratio; CI: confidence interval; *P < 0.05.

### Knockdown of XIST inhibits proliferation, migration and invasion of ESCC cells

**Figure 2:**
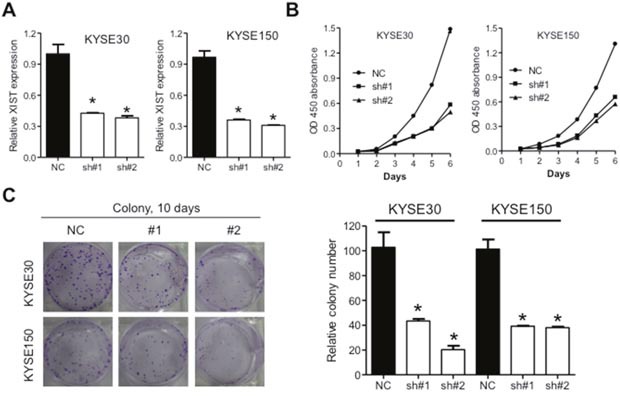
Downregulation of XIST inhibits proliferation of ESCC cells **(A)** Expression of XIST in KYSE30 and KYSE150 cells after transfection with lentivirus containing short hairpins targeting XIST. **(B)** CCK-8 assays indicated that down-regulation of XIST suppressed cell growth *in vitro*. **(C)** Colony formation assays of KYSE30 and KYSE150 cells after knockdown of XIST. Representative images and quantifications were shown. Error bars: mean ± SD, n = 3. *P < 0.05 versus control.

**Figure 3:**
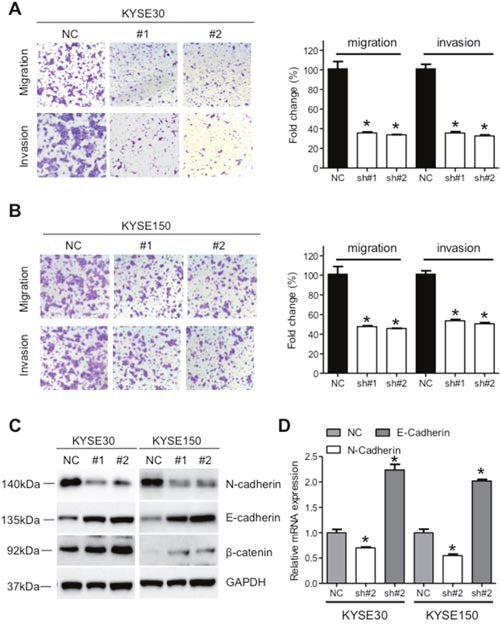
Down-regulation of XIST suppresses migration and invasion of ESCC cells **(A)** Representative images and quantification of migration and invasion of KYSE30 cells after down-regulation of XIST. **(B)** Representative images and quantification of migration and invasion of KYSE150 cells after down-regulation of XIST. **(C)** Expression of E-cadherin, N-cadherin and β-catenin after down-regulation of XIST in KYSE30 and KYSE150 cells. **(D)** mRNA level of E-cadherin and N-cadherin after down-regulation of XIST in KYSE30 and KYSE150 cells. Error bars: mean ± SD, n = 3. *P < 0.05 versus control.

Significant upregulation of XIST in cancer tissues prompted us to investigate its roles on aggressive phenotypes of ESCC cells. KYSE30 and KYSE150 cells with the highest level of XIST were selected for further assays. XIST specific short harpain RNAs (sh#1 and sh#2) and nonspecific short hairpin RNA used as negative control (NC) were transfected into KYSE30 and KYSE150 cells and subsequent qRT-PCR assays confirmed successful knockdown of XIST in ESCC cells (Figure 2A). CCK-8 assays revealed that knockdown of XIST significantly suppressed cell growth in KYSE30 and KYSE150 cells (Figure 2B). Colony formation assays further indicated anti-proliferation activity of XIST knockdown in ESCC cells (Figure 2C). Decreased migration and invasion capacity of KYSE30 and KYSE150 cells was observed after knockdown of XIST (Figure 3A and 3B). As epithelial mesenchymal transition (EMT) initiated metastasis constitutes the major cause of cancer related death [24], we therefore proceed to test whether XIST was involved in EMT of ESCC cells. XIST knockdown resulted in elevated expression of E-cadherin and β-catenin and decreased expression of N-cadherin, indicating EMT underlies the pro-metastasis roles of XIST (Figure 3C and 3D). Altogether, our data indicated that knockdown of XIST inhibits proliferation, migration and invasion of ESCC cells.

### XIST regulates expression of miR-101

**Figure 4:**
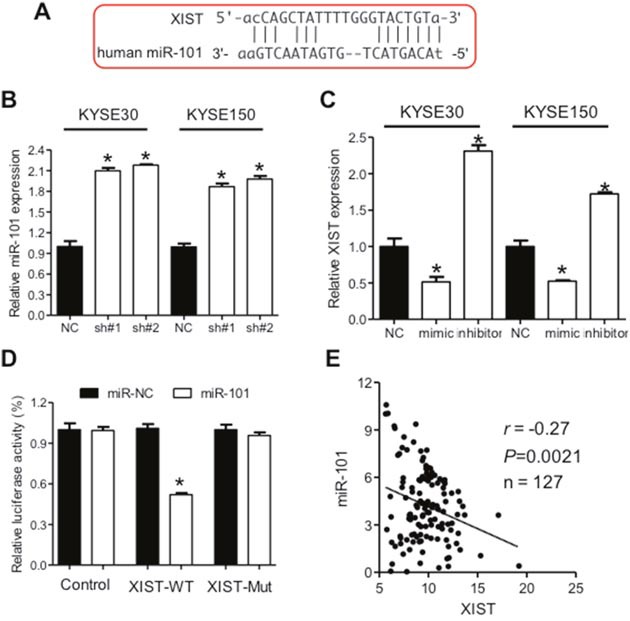
XIST is negatively regulated by miR-101 in ESCC **(A)** Predicted binding sites of miR-101 and XIST. **(B)** Down-regulation of XIST leads to increased miR-101 expression in KYSE30 and KYSE150 cells. **(C)** Expression of XIST after transfection of mimic or inhibitor of miR-101 in KYSE30 and KYSE150 cells. **(D)** Relative luciferase activities of wild type and mutated XIST reporter in HEK293T cells transfected with miR-101 mimic. **(E)** Correlation between expression of XIST and miR-101 in ESCC cancer tissues. Error bars: mean ± SD, n = 3. *P < 0.05 versus corresponding control.

Mountainous evidence are emerging to show that lncRNAs function as competitive endogenous RNA to regulate cell information flow [10] and XIST have been frequently validated to act as molecular sponges for miRNAs [20, 23]. We therefore hypothesized that XIST might facilitate the aggressive phenotypes of ESCC through regulation of miRNA expression. Based on the online database (http://starbase.sysu.edu.cn/), we searched for miRNAs containing complementary bases with XIST and focused on miR-101 (Figure 4A). Increased expression of miR-101 was observed after knockdown of XIST in KYSE30 and KYSE150 cells (Figure 4B). Mimic and inhibitor of miR-101 significantly down- and upregulated expression of XIST in KYSE30 and KYSE150 cells (Figure 4C), respectively. Next, we cloned the wild type (pmirGLO-XIST-WT) and mutated binding site (pmirGLO-XIST-Mut) of miR-101 in the XIST sequence into the reporter vector and employed luciferase assays to confirm the direct relationship between miR-101 and XIST. Overexpression of miR-101 significantly inhibited luciferase activity of pmirGLO-XIST-WT, but not that of pmirGLO-XIST-Mut (Figure 4D). Expression of XIST and miR-101 in 127 ESCC tumor tissues showed an inverse relation (Figure 4E), further confirming that miR-101 may decrease XIST expression in ESCC.

### EZH2 is involved in oncogenic activity of XIST

**Figure 5:**
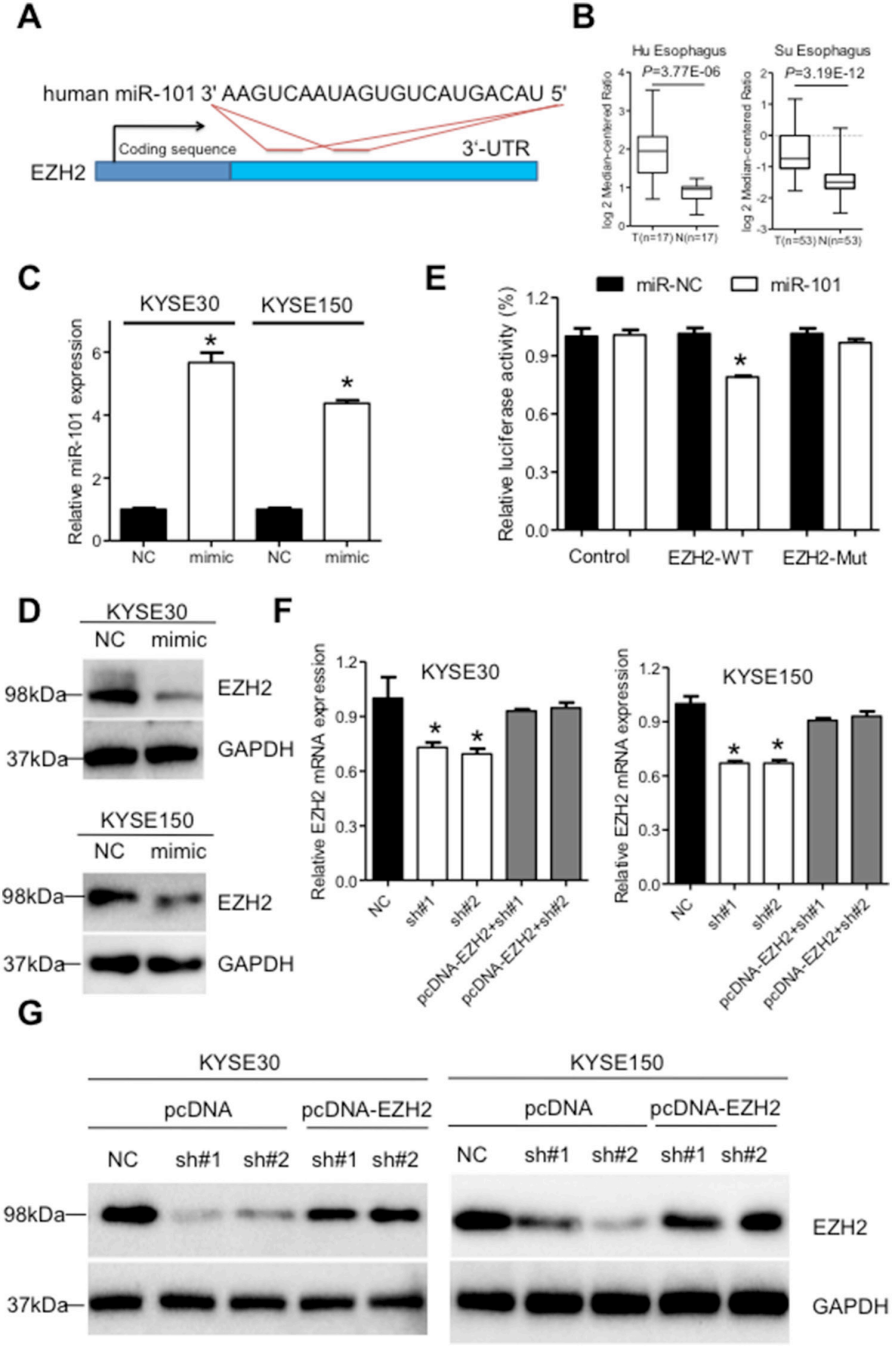
XIST regulates expression of miR-101 targeted gene EZH2 in ESCC **(A)** Predicted target sites of miR-101 in the 3'-UTR of EZH2 gene. **(B)** Expression of EZH2 in ESCC from the Oncomine database (https://www.oncomine.com/). **(C)** Expression of miR-101 in KYSE30 and KYSE150 cells after transfection with mimic of miR-101. **(D)** Expression of EZH2 was detected by western blot in KYSE30 and KYSE150 cells after transfection with miR-101 mimic. **(E)** Relative luciferase activities of wild type and mutated EZH2 3'-UTR reporter in HEK293T cells transfected with miR-101 mimic. **(F)** Relative EZH2 mRNA level in stable XIST knockdown of KYSE30 and KYSE150 cells after transfection with pcDNA-EZH2 lacking the 3'-UTR. **(G)** Western blot analysis of EZH2 expression in stable XIST knockdown of KYSE30 and KYSE150 cells after transfection with pcDNA-EZH2 lacking the 3'-UTR. Error bars: mean ± SD, n = 3. *P < 0.05 versus corresponding control.

**Figure 6:**
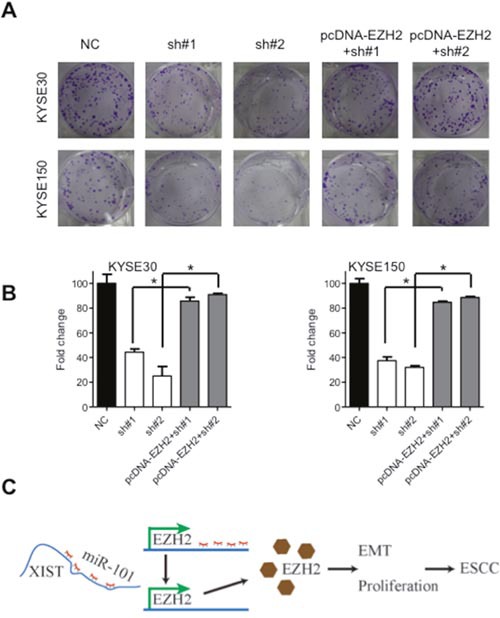
Oncogenic activities of XIST is mediated by EZH2 **(A)** Representative images of colony formation of KYSE30 and KYSE150 cells after downregulation of XIST and co-transfected with the pcDNA-EZH2 lacking the 3'-UTR. **(B)** Quantification of colonies in **(A)**. **(C)** Schematic diagram of dysregulated XIST/miR-101/EZH2 axis in the promotion of ESCC development. Error bars: mean ± SD, n = 3. *P < 0.05 versus corresponding control.

It has been well-established that miRNA could bind to the 3'-untranslated regions (3'-UTR) of protein coding genes and thus regulate their expressions [25]. We prompted to investigate whether aberrant expression of XIST was associated with downstream proteins. Regulative correlations of EZH2 and miR-101 (Figure 5A) have been well documented in several human cancers [25–27] and overexpressed EZH2 predicts poor prognosis of ESCC patients [28]. With the Oncomine database (http://www.oncimine.org/), EZH2 was significantly overexpressed in ESCC tumor tissues compared with that in normal tissues (Figure 5B). Moreover, the protein level of EZH2 was suppressed after overexpression of miR-101 in KYSE30 and KYSE150 cells (Figure 5C and 5D). Luciferase assays further confirmed that miR-101 could bind to the 3'-UTR of EZH2 (Figure 5E). Following qRT-PCR and western blot analysis confirmed that decreased expression of EZH2 in sh#1 and sh#2 cells was attenuated by co-transfection of a vector containing the coding sequence of EZH2, but without the 3'-UTR (Figure 5F and 5G). To gain further insights whether miR-101/EZH2 mediated the oncogenic roles of XIST in ESCC, cell proliferation assays were utilized. As shown in Figure 6A and 6B, reintroduction of the vector containing the coding sequence but lacking the 3'-UTR of EZH2 significantly attenuated the anti-proliferation effects of XIST knockdown in KYSE30 and KYSE150 cells. Additionally, inhibition of EZH2 with GSK126 significantly suppressed cell viability and decreased colony formation of KYSE30 and KYSE150 cells (Supplementary Figure 1A and 1B). In the patient cohort, expression of EZH2 and XIST showed significant positive correlation (Supplementary Figure 2). Collectively, our results indicated that upregulated XIST promotes ESCC malignant phenotype via regulation of miR-101/EZH2 axis (Figure 6C).

### XIST suppressed tumor growth via regulation of miR-101/EZH2 *in vivo*

**Figure 7:**
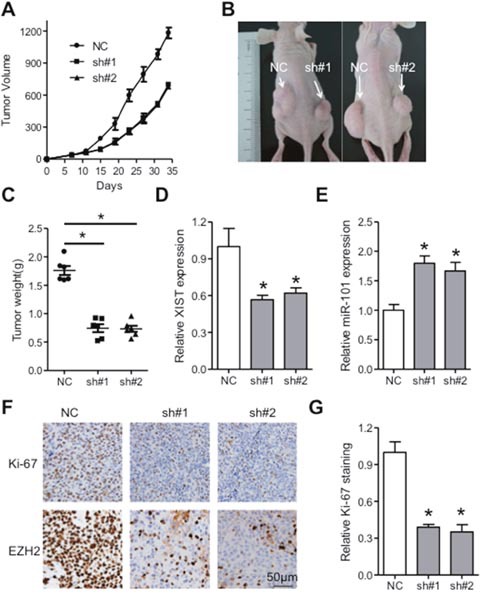
XIST promotes tumor growth of ESCC via regulation of miR-101/EZH2 axis *in vivo* **(A)** Tumor volume of the xenografts in XIST knockdown groups and control group. **(B)** Tumors in nude mice formed in knockdown groups and control group were imaged. **(C)** Weight of dissected tumors in knockdown groups and control group was shown. Expression of XIST **(D)** and miR-101 **(E)** in the dissected tumors of knockdown groups and control group was detected by qRT-PCR. Immunohistochemistry staining **(F)** and quantification analysis of Ki-67 **(G)** and EZH2 in knockdown groups and control group were shown. Scale bars: 50μm. Error bars: mean ± SD, n = 6. *P < 0.05 versus corresponding control.

To gain deeper insights of XIST in ESCC, subcutaneous nude mice model was utilized. KYSE150 cells with stable expression of short hairpin RNAs targeting XIST (sh#1 or sh#2) or scramble sequence (NC) were inoculated into the dorsal flank of nude mice and the tumor volume was monitored. We found that tumors in the knockdown groups grew slower than that in the NC group (Figure 7A-7C). Further analysis indicated that XIST was significantly reduced in the xenografts from the knockdown groups, while expression of miR-101 was elevated compared with that in the control group (Figure 7D and 7E). Moreover, immunohistochemistry analysis of the dissected specimens showed that Ki-67 and EZH2 were significantly decreased in the sh#1 and sh#2 group compared with NC group (Figure 7F and 7G). Altogether, knockdown of XIST suppresses tumor growth via regulation of miR-101/EZH2 axis *in vivo*.

## DISCUSSION

In the present study, we found that XIST was overexpressed in tumor tissues compared with that in normal counterparts and correlated with poor prognosis. Silence of XIST expression significantly inhibited the proliferation, migration and invasion capacity of ESCC cells *in vitro* and suppressed tumor growth *in vivo*. Furthermore, our data provided the first evidence that XIST functions as a molecular sponge for miR-101 and EZH2 and knockdown of XIST exerted tumor-suppressive functions in human ESCC by epigenetic regulation of EZH2 via reciprocal repression of miR-101.

The X chromosome inactivation is essential for equalization of gene expression between males and females during development [29]. XIST plays vital roles in the regulation of X chromosome inactivation [30] and XIST mediated transcription silence requires interaction with SHARP [31] and recruitment of PRC2 via the RNA-binding protein ATRX [32]. Dysregulated expression of XIST was associated with initiation, progression and metastasis of several cancers [12, 13, 31]. For instance, XIST is upregulated in gastric cancer and inhibits expression of EZH2 [20]. Studies have proved that XIST impaired formation of neurospheres of glioblastoma via interaction with miR-152 and KLF2. However, aberrant activation of Akt induced by downregulation of XIST in breast cancer resulted in malignant progression [33]. Yildirim E et al. found that mice with deleted XIST expression developed highly aggressive hematopoietic disorders, indicating tumor suppressive roles of XIST [15]. Combined these studies with our data, we speculated that XIST may play distinct roles depending on the tumor type and may interfere with different miRNAs in different tumors.

EZH2 constitutes the main component of PRC2 as a transcriptional repressor [34] and dysregulation of EZH2 is implicated in progression of many types of human cancers [35, 36] including ESCC [28]. Studies also confirmed the post-transcriptional regulation mechanisms underlying EZH2 expression [26, 37] and these include miR-101 in gastric [20], hepatocellular [27] and esophageal cancer [25]. Our studies revealed that knockdown of XIST resulted in downregulation of EZH2 and reintroduction of EZH2 without the binding site for miR-101 significantly attenuated the anti-proliferation effects after knockdown of XIST, which is in concordance with a recent report demonstrating oncogenic roles of XIST in gastric cancer [19].

Collectively, our data provided the first evidence that upregulated expression of XIST could promote malignant progression of ESCC by modulating the miR-101/EZH2 axis and might be used as therapeutic target of ESCC.

## MATERIALS AND METHODS

### Patient samples

The ESCC cancer tissues and pair-matched normal epithelial squamous tissues utilized in this study (collected postoperatively from 2007 to March 2009) were obtained from patients who underwent radical resections at Sun Yat-sen University Cancer Center. None of them received preoperative chemo- or radiotherapy. Our study was approved by the Ethics Committee of the Sun Yat-sen University Cancer Center and written informed consent was obtained from all patients. The specimens were immediately frozen in liquid nitrogen and stored at −80°C until RNA extraction. Complete clinicopathologically and follow-up data of these patients were collected from the medical record.

### Cell lines and cell culture

Six esophageal squamous cell carcinoma cell lines (KYSE30, KYSE510, KYSE410, KYSE520, KYSE140 and KYSE150) were purchased from the Deutsche Sammlung von Mikroorganismen und Zellkulturen (DSMZ, Braunschweig, Germany). All cells were cultured in Dulbecco's Modified Essential Medium (DMEM) medium (Hyclone, USA) supplemented with 10% fetal bovine serum (10% FBS), and maintained in a humidified incubator at 37°C with 5% CO_2_.

### RNA extraction and qRT-PCR

Total RNA was extracted from patient samples and cells using Trizol reagent (Invitrogen, Carlsbad, CA, USA) according to the manufacturer's protocol. The RNA was transcribed to cDNA using the Reverse Transcription Kit (Takara, Dalian, China). qRT-PCR was performed with TaqMan Mix using ABI 7500 fast real-time PCR system (Applied Biosystems, Darmstadt, Germany). GAPDH was employed as the internal control for both mRNA and lncRNA. The primers used for qRT-PCR assays were obtained from Life Technology and summarized in Supplementary Table 1. TaqMan miRNA assays (Applied Biosystems) were used to detect miRNA expression according to the manufacturer's instructions. The qRT-PCR reactions were performed in triplicate. The relative expression of RNAs was calculated using the 2^−ΔΔCt^ method.

### Lentivirus and cell transfection

Lentivirus particles expressing short hairpin RNA (shRNA) targeting XIST or scrambled oligonucleotides (NC) were purchased from GenePharma (Shanghai, China). The KYSE30 and KYSE150 cells were transfected with lentivirus. At 48h after transfection, cells were treated with puromycin (3μg/ml) for 7 days and the transfection efficiency was determined by GFP-positive cell percentage.

The miR-101 mimic, miR-101 inhibitor and negative control (miR-NC) were obtained from Ribobio (Guangzhou, China). To restore EZH2 expression, a pcDNA3.1-EZH2 plasmid, which contained the coding sequences but lacked the 3'-UTR of EZH2 was constructed. The cell transfection was performed with Lipofectamine2000 according to the manufacturer's instructions. Briefly, cells were placed in a 6-well plate the day before transfection. At 48h after transfection, cells were collected for subsequent analysis or subjected to qRT-PCR and western blot assays to confirm the transfection efficiency.

### Cell proliferation and colony formation assays

For the proliferation assays, cells were plated in 96-well plates at a density of 5×10^3^/ml. Cell proliferation was assessed using the Cell Counting Kit-8 (CCK-8, Dojindo, Tokyo, Japan) according to the manufacturer's protocol. Each group contains five wells and all experiments were replicated thrice. For colony formation assay, Five hundred cells were plated into 6-well plates and incubated in DMEM with 10% FBS at 37°C for 10 days. Then the cells were fixed with polyoxymethylene and stained with 0.1% crystal violet and the colonies were counted. For drug treatment, GS126 (Selleck, Huston, USA) at indicated concentrations was added into culture medium and cell viability and colony formation were examined.

### *In vitro* migration and invasion assays

The *in vitro* migration assays were performed with the 24-well chambers (Corning, USA). Indicated cells were resuspended in 200μl serum-free medium at a density of 5×10^5^ cells/ml and seeded into the upper chamber with (invasion) or without (migration) matrigel, while 500μl medium containing 50% FBS was placed in the lower chamber as chemo attractant. After incubation at 37°C for 48h, cells were fixed with polyoxymethylene, stained with 0.1% crystal violet. After scraping of cells remaining on the upper chamber by cotton swab, those migrated or invaded into the lower chamber were photographed and five fields were counted under a microscope.

### Western blot and immunohistochemical analysis

Cells were collected, rinsed with cold PBS twice and then lysed in RIPA buffer with protease inhibitor (Selleck, Houston, USA) on the ice. After centrifugation, the supernatant was subjected to BCA assays (Thermo Fisher, USA). Equal quantity of proteins was then separated by SDS-PAGE gel and transferred to PVDF membrane. 5% nonfat milk dissolved in Tris-buffered saline (TBS) containing 0.1% Tween-20 was used as block buffer and the membranes were incubated with primary antibodies at 4°C overnight with gentle shake. The proteins in the membranes were visualized with the SuperSignal^®^ ECL Kit (Pierce, USA). The immunohistochemical assays were conducted according to a previous report [24].

### Luciferase reporter assay

The XIST fragment containing the putative miR-101 binding site was cloned into a pmirGlO dual-luciferase miRNA Target Expression Vector (Promega, Madison, WI, USA) to form the reporter vector named as pmirGLO-XIST-WT. The same vector containing the mutated site for miR-101 in XIST sequence was named as pmirGLO-XIST-Mut. Luciferase reporter assay was performed as described previously [23]. Briefly, ESCC cells were transfected in 48-well plates with 200 ng firefly luciferase plasmids, 4 ng control Renilla luciferase vector pRL-TK, together with other vectors including miR-101 mimics or miR-NC and pmirGLO-XIST-WT or pmirGLO-XIST-Mut. At 48h after transfection, luciferase activity was measured using a Dual-Luciferase Reporter Assay System (Promega, Madison, WI, USA) according to the manufacturer's protocol.

### Tumor formation assays in a nude mouse model

Female BABL/c nude mice (four to five weeks old) were purchased from the Beijing Vital River Laboratory Animal Technology Co., Ltd. KYSE150 cells (1×10^6^) stably expressing shRNA targeting XIST or scramble sequence were subcutaneously injected into the dorsal flank of BALB/C nude mice. The tumor volumes were monitored twice every week after injection. All mice were sacrificed 35 days afterward, and the xenografts were dissected out for qRT-PCR or immunohistochemical analysis. All the animal experiments were performed according to the National Institutes of Health animal use guidelines on the use of experimental animals. The protocol was approved by the Committee on the Ethics of Animal Experiments of Sun Yat-sen University Cancer Center.

### Statistical analysis

Experimental data in our study were presented as means ± standard deviation (SD). Statistical analyses were performed using the SPSS software package (version 23.0, SPSS Inc.) or GraphPad Prism 6.0 (GraphPad Software Inc., San Diego, CA, USA) with the Student's t-test or one-way ANOVA. The chi-square test or Fisher's exact test was used to analyze the association of XIST expression with clinicopathological parameters. Survival curves were generated using the Kaplan-Meier method and assessed using the log-rank test. The Cox proportional hazard regression model was performed to identify independent prognostic factors. The relationship between the expression of XIST and miR-101 in the ESCC cancer tissues was analyzed with Pearson's correlation. *P* < 0.05 was considered to be statistically significant.

## SUPPLEMENTARY MATERIALS FIGURES AND TABLE



## References

[1] Torre LA, Bray F, Siegel RL, Ferlay J, Lortet-Tieulent J, Jemal A (2015). Global cancer statistics, 2012. CA Cancer J Clin.

[2] Chen W, Zheng R, Baade PD, Zhang S, Zeng H, Bray F, Jemal A, Yu XQ, He J (2016). Cancer statistics in China, 2015. CA Cancer J Clin.

[3] Rustgi AK, El-Serag HB (2014). Esophageal carcinoma. N Engl J Med.

[4] Markar SR, Wiggins T, Ni M, Steyerberg EW, Van Lanschot JJ, Sasako M, Hanna GB (2015). Assessment of the quality of surgery within randomised controlled trials for the treatment of gastro-oesophageal cancer: a systematic review. Lancet Oncol.

[5] Demeester SR (2009). Epidemiology and biology of esophageal cancer. Gastrointest Cancer Res.

[6] Wang B, Yin BL, He B, Chen C, Zhao M, Zhang W, Xia ZK, Pan Y, Tang J, Zhou X, Yin N (2012). Overexpression of DNA damage-induced 45 alpha gene contributes to esophageal squamous cell cancer by promoter hypomethylation. J Exp Clin Cancer Res.

[7] Casale V, Lapenta R, Gigliozzi A, Villotti G (1999). Endoscopic palliative therapy in neoplastic diseases of the esophagus. J Exp Clin Cancer Res.

[8] Song H, Xu B, Yi J (2012). Clinical significance of stanniocalcin-1 detected in peripheral blood and bone marrow of esophageal squamous cell carcinoma patients. J Exp Clin Cancer Res.

[9] Hanahan D, Weinberg RA (2011). Hallmarks of cancer: the next generation. Cell.

[10] Batista PJ, Chang HY (2013). Long noncoding RNAs: cellular address codes in development and disease. Cell.

[11] Sun M, Kraus WL (2015). From discovery to function: the expanding roles of long noncoding RNAs in physiology and disease. Endocr Rev.

[12] Briggs SF, Reijo Pera RA (2014). X chromosome inactivation: recent advances and a look forward. Curr Opin Genet Deve.

[13] Cerase A, Pintacuda G, Tattermusch A, Avner P (2015). Xist localization and function: new insights from multiple levels. Genome Biol.

[14] Schmitt AM, Chang HY (2016). Long noncoding RNAs in cancer pathways. Cancer Cell.

[15] Yildirim E, Kirby JE, Brown DE, Mercier FE, Sadreyev RI, Scadden DT, Lee JT (2013). Xist RNA is a potent suppressor of hematologic cancer in mice. Cell.

[16] Wu X, Lim ZF, Li Z, Gu L, Ma W, Zhou Q, Su H, Wang X, Yang X, Zhang Z (2017). NORAD expression is associated with adverse prognosis in esophageal squamous cell carcinoma. Oncol Res Treat.

[17] Lee JT (2011). Gracefully ageing at 50, X-chromosome inactivation becomes a paradigm for RNA and chromatin control. Nat Rev Mol Cell Biol.

[18] Jeon Y, Lee JT (2011). YY1 tethers Xist RNA to the inactive X nucleation center. Cell.

[19] Zhao J, Sun BK, Erwin JA, Song JJ, Lee JT (2008). Polycomb proteins targeted by a short repeat RNA to the mouse X chromosome. Science.

[20] Chen DL, Ju HQ, Lu YX, Chen LZ, Zeng ZL, Zhang DS, Luo HY, Wang F, Qiu MZ, Wang DS, Xu DZ, Zhou ZW, Pelicano H (2016). Long non-coding RNA XIST regulates gastric cancer progression by acting as a molecular sponge of miR-101 to modulate EZH2 expression. J Exp Clin Cancer Res.

[21] Wassef M, Margueron R (2017). The multiple facets of PRC2 alterations in cancers. J Mol Biol.

[22] Fang J, Sun CC, Gong C (2016). Long noncoding RNA XIST acts as an oncogene in non-small cell lung cancer by epigenetically repressing KLF2 expression. Biochem Biophys Res Commun.

[23] Yao Y, Ma J, Xue Y, Wang P, Li Z, Liu J, Chen L, Xi Z, Teng H, Wang Z, Li Z, Liu Y (2015). Knockdown of long non-coding RNA XIST exerts tumor-suppressive functions in human glioblastoma stem cells by up-regulating miR-152. Cancer Lett.

[24] Nieto MA, Huang RY, Jackson RA, Thiery JP (2016). Emt: 2016. Cell.

[25] Lin C, Huang F, Li QZ, Zhang YJ (2014). miR-101 suppresses tumor proliferation and migration, and induces apoptosis by targeting EZH2 in esophageal cancer cells. Int J Clin Exp Pathol.

[26] Huang SD, Yuan Y, Zhuang CW, Li BL, Gong DJ, Wang SG, Zeng ZY, Cheng HZ (2012). MicroRNA-98 and microRNA-214 post-transcriptionally regulate enhancer of zeste homolog 2 and inhibit migration and invasion in human esophageal squamous cell carcinoma. Mol Cancer.

[27] Xu L, Beckebaum S, Iacob S, Wu G, Kaiser GM, Radtke A, Liu C, Kabar I, Schmidt HH, Zhang X, Lu M, Cicinnati VR (2014). MicroRNA-101 inhibits human hepatocellular carcinoma progression through EZH2 downregulation and increased cytostatic drug sensitivity. J Hepatol.

[28] Yamada A, Fujii S, Daiko H, Nishimura M, Chiba T, Ochiai A (2011). Aberrant expression of EZH2 is associated with a poor outcome and P53 alteration in squamous cell carcinoma of the esophagus. Int J Oncol.

[29] Payer B, Lee JT (2008). X chromosome dosage compensation: how mammals keep the balance. Ann Rev Genet.

[30] Clemson CM, McNeil JA, Willard HF, Lawrence JB (1996). XIST RNA paints the inactive X chromosome at interphase: evidence for a novel RNA involved in nuclear/chromosome structure. J Cell Biol.

[31] McHugh CA, Chen CK, Chow A, Surka CF, Tran C, McDonel P, Pandya-Jones A, Blanco M, Burghard C, Moradian A, Sweredoski MJ, Shishkin AA, Su J (2015). The Xist lncRNA interacts directly with SHARP to silence transcription through HDAC3. Nature.

[32] Sarma K, Cifuentes-Rojas C, Ergun A, Del Rosario A, Jeon Y, White F, Sadreyev R, Lee JT (2014). ATRX directs binding of PRC2 to Xist RNA and polycomb targets. Cell.

[33] Huang YS, Chang CC, Lee SS, Jou YS, Shih HM (2016). Xist reduction in breast cancer upregulates AKT phosphorylation via HDAC3-mediated repression of PHLPP1 expression. Oncotarget.

[34] Debeb BG, Gong Y, Atkinson RL, Sneige N, Huo L, Gonzalez-Angulo AM, Hung MC, Valero V, Ueno NT, Woodward WA (2014). EZH2 expression correlates with locoregional recurrence after radiation in inflammatory breast cancer. J Exp Clin Cancer Res.

[35] Wu J, Zhao S, Tang Q, Zheng F, Chen Y, Yang L, Yang X, Li L, Wu W, Hann SS (2015). Activation of SAPK/JNK mediated the inhibition and reciprocal interaction of DNA methyltransferase 1 and EZH2 by ursolic acid in human lung cancer cells. J Exp Clin Cancer Res.

[36] D'Angelo V, Iannotta A, Ramaglia M, Lombardi A, Zarone MR, Desiderio V, Affinita MC, Pecoraro G, Di Martino M, Indolfi P, Casale F, Caraglia M (2015). EZH2 is increased in paediatric T-cell acute lymphoblastic leukemia and is a suitable molecular target in combination treatment approaches. J Exp Clin Cancer Res.

[37] Kottakis F, Polytarchou C, Foltopoulou P, Sanidas I, Kampranis SC, Tsichlis PN (2011). FGF-2 regulates cell proliferation, migration, and angiogenesis through an NDY1/KDM2B-miR-101-EZH2 pathway. Mol Cell.

