# Supplementary material for: Long noncoding RNA XIST promotes malignancies of esophageal squamous cell carcinoma via regulation of miR-101/EZH2
Source: Oncotarget. 2017 Jun 27;8(44):76015–28. doi: 10.18632/oncotarget.18638 (PMC5652682; doi:10.18632/oncotarget.18638)
Supplement: Supplementary file 1 [file oncotarget-08-76015-s001.pdf]

Long noncoding RNA XIST promotes malignancies of esophageal squamous cell carcinoma via regulation of miR-101/EZH2

SUPPLEMENTARY MATERIALS

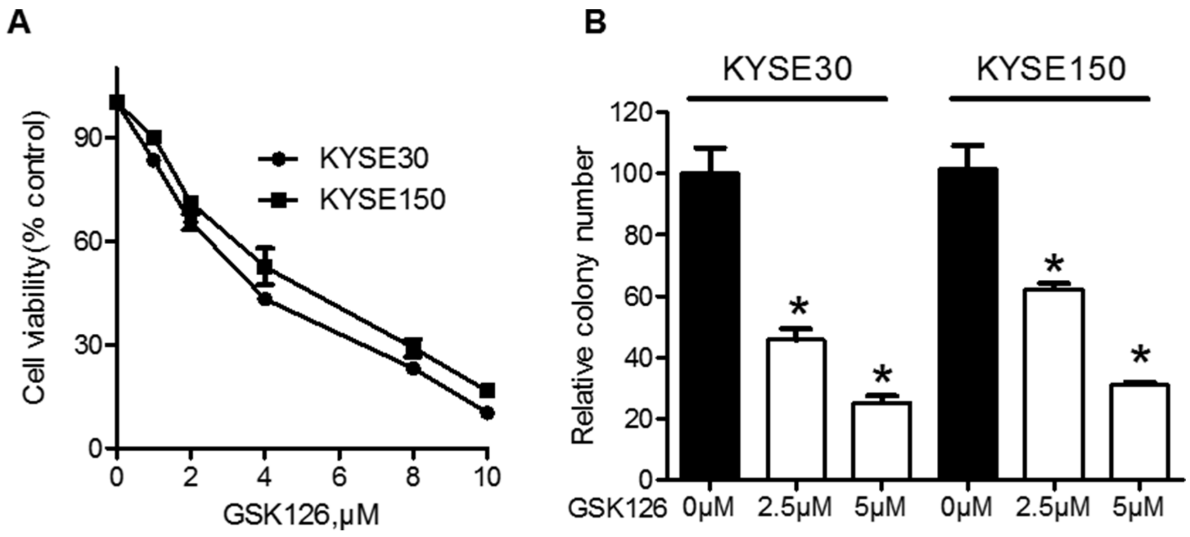

Supplementary Figure 1: Inhibition of EZH2 suppressed cell growth and decreased colony formation. (A) KYSE30 and KYSE150 cells were exposure to GSK126 at various concentrations for 8 days and cell viability were measured with CCK-8 assays. Cell viability (%) was normalized using cells treated with 0μM. **(B)** Colony formation assays of KYSE30 and KYSE150 cells treated with GSK126 at 2.5μM and 5μM. Error bars: mean ± SD, n = 3. **P* < 0.05 versus control.

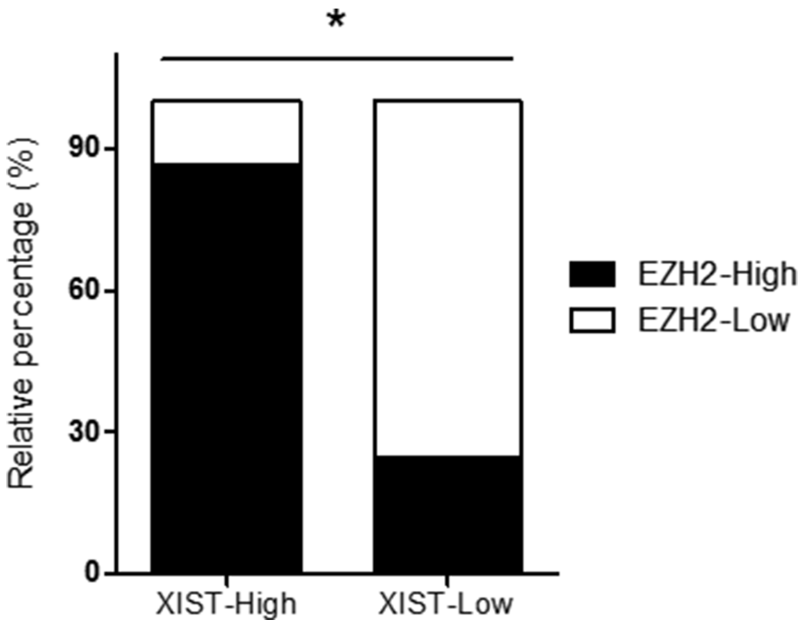

Supplementary Figure 2: Expression of EZH2 correlated positively with XIST in ESCC patient samples. Histogram of EZH2 expression according to immunohistochemical assays in XIST high and low group. *$P < 0.05$ with chi-square tests.

Supplementary Table 1: Primers sequence for qPCR

Gene	Sequence (5'-3')
XIST-F	CTCTCCATTGGGTTCAC
XIST-R	GCGGCAGGTCTTAAGAGATGAG
E-Cadherin -F	CGAGAGCTACACGTTCACGG
E-Cadherin -R	GGGTGTCGAGGGAAAAATAGG
N-Cadherin -F	TCAGGCGTCTGTAGAGGCTT
N-Cadherin -R	ATGCACATCCTTCGATAAGACTG
GAPDH-F	ATCACCATCTTCCAGGAGCGA
GAPDH-R	CCTTCTCCATGGTGGTGAAGAC
